# Physiological Performance Measures as Indicators of CrossFit^®^ Performance

**DOI:** 10.3390/sports7040093

**Published:** 2019-04-22

**Authors:** Joshua D. Dexheimer, E. Todd Schroeder, Brandon J. Sawyer, Robert W. Pettitt, Arnel L. Aguinaldo, William A. Torrence

**Affiliations:** 1 Department of Exercise Science, Concordia University Chicago, Riverforest, IL 60305, USA; William.Torrence@cuchicago.edu; 2 Department of Kinesiology, Azusa Pacific University, Azusa, CA 91702, USA; 3 Division of Biokinesiology & Physical Therapy, University of Southern California, Los Angeles, CA 90033, USA; eschroed@usc.edu; 4 Department of Kinesiology, Point Loma Nazarene University, San Diego, CA 92106, USA; bsawyer@pointloma.edu (B.J.S.); arnelaguinaldo@pointloma.edu (A.L.A.); 5 Office of Research and Sponsored Projects, Rocky Mountain University of Health Professions, Provo, UT 84606, USA; rpettitt@rmuohp.edu

**Keywords:** CrossFit^®^ sport performance, physiological indicators, benchmark performance, VO_2max_, critical speed, D′, strength

## Abstract

CrossFit^®^ began as another exercise program to improve physical fitness and has rapidly grown into the “sport of fitness”. However, little is understood as to the physiological indicators that determine CrossFit^®^ sport performance. The purpose of this study was to determine which physiological performance measure was the greatest indicator of CrossFit^®^ workout performance. Male (*n* = 12) and female (*n* = 5) participants successfully completed a treadmill graded exercise test to measure maximal oxygen uptake (VO_2max_), a 3-minute all-out running test (3MT) to determine critical speed (CS) and the finite capacity for running speeds above CS (D′), a Wingate anaerobic test (WAnT) to assess anaerobic peak and mean power, the CrossFit^®^ total to measure total body strength, as well as the CrossFit^®^ benchmark workouts: Fran, Grace, and Nancy. It was hypothesized that CS and total body strength would be the greatest indicators of CrossFit^®^ performance. Pearson’s r correlations were used to determine the relationship of benchmark performance data and the physiological performance measures. For each benchmark-dependent variable, a stepwise linear regression was created using significant correlative data. For the workout Fran, back squat strength explained 42% of the variance. VO_2max_ explained 68% of the variance for the workout Nancy. Lastly, anaerobic peak power explained 57% of the variance for performance on the CrossFit^®^ total. In conclusion, results demonstrated select physiological performance variables may be used to predict CrossFit^®^ workout performance.

## 1. Introduction

CrossFit^®^ (CrossFit, Inc., Washington, DC, USA) began as another exercise program to improve physical fitness and has grown exponentially from 49 CrossFit^®^ gym affiliates in 2005 to over 13,000 today [1,2]. CrossFit^®^ training prescribes “constantly varied, high intensity, functional movement”, with functional movement defined as compound multi-joint exercises [2]. CrossFit^®^ training has also been described as a form of high-intensity functional training (HIFT) which has been defined as “a training style [or program] that incorporates functional, multimodal movements, performed at relatively high intensity, and designed to improve parameters of general physical fitness and performance” [2,3]. Though all affiliates are different, a CrossFit^®^ class may consist of a warm-up, skill or strength exercise, and workout of the day (multiple modes of exercise performed in a circuit fashion) all completed within an hour. 

The CrossFit^®^ training paradigm may prove beneficial in improving health and physiological performance measures. Those that participated in CrossFit^®^ training revealed improvements in strength, aerobic and anaerobic capacity, and power output [4,5,6]. CrossFit^®^ training has also improved the following health variables: Body composition, diastolic blood pressure, and resting heart rate [6,7]. These same variables and measures have been significantly correlated to improved physical performance and may play a role in CrossFit^®^ sport performance [8,9,10]. As CrossFit^®^ has evolved as a training program and into the “sport of fitness”, further investigation of the physiological variables that influence CrossFit^®^ sport performance is warranted [2,3,11].

The rise in athletes competing in CrossFit^®^ competitions has grown exponentially. Athletes of all levels that compete aim to complete a workout as fast as possible for time, for as many repetitions as possible in an allotted time, or for maximal weight lifted. Athletes are monitored by judges to ensure competition standards are met and earn points based on their place of finish. Participation in the CrossFit^®^ Open, an online qualifier competition open to athletes of all levels, has increased from 26,000 in 2011 to over 300,000 in 2016 [12]. Through the CrossFit^®^ Open and other sanctioned CrossFit^®^ competitions, the top 40 men and 40 women in the world then go on to compete as individuals at the CrossFit^®^ Games. As demonstrated by the number of participants in the CrossFit^®^ Open, the majority of athletes that compete in CrossFit^®^ would be considered amateurs or recreational athletes. 

Given that these individuals who participate in CrossFit^®^ competitions exercise for sport, we hypothesize there may be physiological indicators of CrossFit^®^ performance. However, only a couple of studies have attempted to find predictors of CrossFit^®^ performance in amateur or recreational athletes, and these showed conflicting results [13,14]. One study revealed experience and age alongside the physiological variables aerobic capacity and anaerobic power significantly influenced CrossFit^®^ performance [13]. The only other study to examine physiological performance measures predicting CrossFit^®^ performance revealed strength to be the only significant predictor of two workouts [14]. This conflicts with findings in the previous study [13]. The varied results, limitations, and suggestions for future research of each study have provided a starting point for further investigations to clarify physiological indicators of CrossFit^®^ sport performance. 

CrossFit^®^ athletes must sustain high-intensity exercise during workouts to beat competitors. Therefore, a maximal sustainable pace may be required. Critical speed (CS) is mathematically defined as the speed-asymptote of the hyperbolic relationship between speed and time-to-exhaustion [15]. CS has also demonstrated a significant relationship to the respiratory compensation point and, when expressed as speed or metabolic rate, was not significantly different [16]. Thus, CS is a high-intensity sustainable speed that may indicate performance during a CrossFit^®^ workout [17]. Further examination of physiological variables that may predict CrossFit^®^ sport performance may inform how well an athlete will perform on certain workouts. Given that the study is examining amateur/recreational athletes, recognizing the physiological variables that best predict CrossFit^®^ sport performance may also inform training strategies to improve those physiological variables. Therefore, the purpose of this study was to determine which physiological variables were the best indicators of CrossFit^®^ performance in four CrossFit^®^ benchmark workouts performed by amateur or recreational CrossFit^®^ athletes. We hypothesized that total body strength measured from the CrossFit^®^ total and CS from the 3-minute all-out test (3MT) would be the greatest indicators of CrossFit^®^ performance. 

## 2. Methods

### 2.1. Participants

An a priori power analysis was computed using G*Power 3.1 [18]. CS and total body strength were hypothesized to be the most significant indicators of CrossFit^®^ performance. Power was calculated from CS. However, no study has examined CS as a predictor of CrossFit^®^ performance. Thus, a significant variable most closely associated with CS from a previous study was used, VO_2_ at anaerobic threshold [14]. The lowest significant correlation of VO_2_ at anaerobic threshold (r = −0.53) was used for the power analysis. Inputting 0.53 as the correlation coefficient and setting the alpha level of significance at 0.05 and power to 0.8 a sample size of 25 participants was calculated. 

Thirty-seven individuals were recruited from a local CrossFit^®^ gym. Two extreme values were identified using a box plot, and Cook’s distance values revealed they may have undue influence on the results; thus, two participants were excluded from the analysis. Participants were not restricted from continuing their training or recreational activities. Due to this, four participants did not complete the study due to an injury unrelated to the study. Two participants dropped out due to conflicting time commitments. The remaining participants were excluded due to one of the following: (1) An equipment limitation of not being able to provide enough resistance for the Wingate anaerobic test (WAnT), (2) Pacing during the 3MT, or (3) 3% verification phase (VP) was not met on the graded exercise test (GXT). By the end of data collection, a total of 17 participants were included in the final analysis. Participants were between the ages of 18–45 having over 1 year of exercise training experience that would allow them to be familiar with and have the ability to perform the CrossFit^®^ benchmark workouts without modifications or scaling. Though participants were not assessed on their familiarity with the benchmark workouts, we did ensure familiarity with lifts and exercise that are common-place with CrossFit^®^ participation. Normative data collected on CrossFit^®^ benchmark workout performance indicated participants in the current study ranked around the 50th percentile [19]. 

All participants gave written consent for the study, which was approved by the Point Loma Nazarene University and Concordia University of Chicago Institutional Review Board. Participant characteristics are presented in Table 1.

### 2.2. Protocol

Data collection took place at Point Loma Nazarene University and CrossFit^®^ Fortius^®^. Data collection occurred over the course of 7 visits. Given that each test and workout required exercising at maximal and high-intensities, a minimum of 48 h rest was required between tests, workouts, or training, as well as having fasted for 2–3 h and with no caffeine. Participants completed each test and workout (described below) within a 4-week time span, with the tests and workouts counterbalanced to avoid an order-effect.

### 2.3. Body Composition and Anthropometrics

Each participant underwent a series of descriptive measurements evaluating body composition and anthropometrics. Height, which represents the perpendicular distance between the top of the head and the bottom of the feet, was measured barefoot, using a stadiometer (Seca Inc. Hamburg, Germany). Body composition was assessed using air displacement plethysmography via the BodPod (Cosmed, Concord, CA, USA). The BodPod was calibrated daily before testing according to the manufacturer’s instructions. This device has demonstrated to be both a valid and reliable measurement of body composition [20,21]. Standard testing procedures were followed, and body fat percentage was calculated using Siri’s formula [20].

### 2.4. Graded Exercise Test

All participants performed an individualized custom treadmill GXT. Pulmonary ventilation and gas exchange were measured continuously with two Parvo Medics TrueOne 2400 (Parvo Medics, Sandy, UT, USA) units, while heart rate was measured with a Polar heart rate monitor (Polar, Lake Success, NY, USA). Two units were used due to the high volume of testing, and previous findings reported low inter-unit errors of 1.5–2.1% [22]. Standard flowmeter and gas calibrations were performed before each test. After collecting resting data for 2 min, subjects began a 5-minute warm-up walking between 3.0–3.5 mph. Following the warm-up phase, the individualized custom treadmill GXT commenced. To create a custom GXT protocol, a prediction VO_2max_ was performed by using a non-exercise regression equation and then deriving a speed estimate with that metabolic value [23]. The projected peak was then divided by the number of stages to yield a GXT duration within 8–12 min [23]. All participants started at 5 mph with a 3% grade, and each minute, the speed increased with the grade remaining constant. Investigators used verbal encouragement as a form of extrinsic motivation to motivate participants to their maximum effort. VO_2max_ was obtained by the average of the two-highest consecutive 15-second oxygen uptake averages during the test. To verify attainment of VO_2max_, participants actively recovered for 5–10 min walking between 1.5–3.5 mph, then each subject performed a supramaximal square-wave verification test at 105% of the speed, with the same grade, obtained during the maximal exercise test until exhaustion [24]. VO_2max_ measured during the VP was then compared to VO_2max_ on the ramp test. GXT VO_2max_ and VP VO_2max_ had to be within 3% for the GXT VO_2max_ to be accepted as a true max. If participants VO_2max_ and VO_2_ verification differed greater than 3%, participants came back to perform the test again on the same unit, or their results were excluded from analysis. 

Using results from the GXT, we were able to identify the speed evoking gas exchange threshold (GET) and VO_2max_ using a linear interpolation method [25]. With a GXT, the physiological response to a given change in speed is not instantaneous but delayed, typically by 1 min. Therefore, an intensity evoking a specific gas exchange value is associated with the specific intensity preceding the measurement by 1 min. This was calculated as the speed (mph) = incremental stage change value divided by four as data were collected every 15 s and stage speed increased each minute. These calculations were used to calculate the average of the speed at GET and VO_2max_ (50 %Δ) to confirm CS results from the 3MT [26].

### 2.5. 3-Minute All-Out-Test

For the 3MT, participants first performed a standardized general and dynamic warm-up created by a certified strength and conditioning specialist. Then, participants ran on a flat track as fast as possible for 3 min and 5 s using a GPS tracking app, Sports Tracker (Amer Sports Digital Services Oy, Vantaa, Finland), which was used to derive CS and D′. The 3MT presumes that a runner will expend D′, the finite capacity for running speeds above CS, within 2.5 min of all-out effort whereby the mean speed between 2.5 and 3.0 minute will reach a nadir at CS [26]. Thus, D′ from a running 3MT was derived using:

D′ = t (S150s − CS)

where time (t) equals 150 s, S150s (m/s) equals the average speed for the first 150 s, and CS (m/s) is the average speed between 150 s and 180 s [26]. 

Utilizing results from the GXT, 50 %Δ, pacing was detected during a 3MT. Meaning, a CS significantly exceeding 50 %Δ would denote an inflated CS from pacing and warranted retesting or exclusion of those data. Given that the GXT protocol entails running at a constant 3% grade, a series of regression equations were utilized to convert treadmill speed and grade to equivalent outdoor running speed. 

### 2.6. Wingate Anaerobic Test (WAnT)

To assess mean and peak anaerobic power, as well as fatigue index (FI), participants performed a 30-second WAnT. The WAnT was completed on a Monark 839e (Monark Exercise AB, Sweden) mechanically braked cycle ergometer with resistance set to 7.5% of the subject’s body weight [27,28]. Participants were first fitted to a comfortable seat height with approximately 5–10 degrees of knee flexion. Participants began with a 5-minute warm-up with 3, 4–6 s high revolution spins [27]. A 10-second countdown was initiated in which the participant began to pedal as fast as possible, and then resistance was added [27]. The participants pedaled as hard and fast as possible for the entire 30 s in which peak power (highest 5-second average wattage), mean power (average 30-second wattage), and FI were calculated [27,28]. Power data were collected in Watts using the Monark 839E Analysis PC Software (Monark Exercise AB, Sweden).

### 2.7. Total Body Strength—CrossFit^®^ Total

To assess maximal strength, participants had 60 min to complete the CrossFit^®^ total (see Reference [29] for movement standards): 1-repetition maximum (RM) back squat, strict shoulder-press, and deadlift [29]. Load percentages were provided, and participants had 3–5 attempts to complete a max 1RM lift. Individual and combined lift totals were recorded. 

### 2.8. CrossFit^®^ Workouts

Participants performed the CrossFit^®^ benchmark workouts (Grace, Fran, and Nancy) to determine CrossFit^®^ performance. Participants aimed to complete each workout as fast as possible. Coaches signed and collected a scoresheet verifying the participant completed the workouts correctly and at the respective time. 

Fran is a CrossFit^®^ benchmark workout in which participants completed 3 rounds of 21-15-9 repetitions of thrusters and pull-ups. The thruster technique consisted of a front squat, in which the hips descended below the knees, into a push-press, ending with the knees, hips, and elbows in full extension, using a barbell (95lb men/65lb women). Participants were able to perform either strict, kipping, or butterfly pull-ups as long as the chin went above the bar. The entire workout was as follows: 21 thrusters, 21 pull-ups, 15 thrusters, 15 pull-ups, 9 thrusters, and 9 pull-ups for time.

Grace is a CrossFit^®^ benchmark workout consisting of 30 clean and jerks (135lb men/95lb women). With each repetition starting from the floor, participants performed either a power clean or squat clean and then completed any type of shoulder to overhead jerk movement: Power jerk or split jerk. The rep was completed upon both feet returning to center with full hip, knee, and elbow extension. 

Nancy is a CrossFit^®^ benchmark workout consisting of 5 rounds of a 400 m run and 15 overhead squats, in which the hips descended below the knees and ascended to full hip and knee extension, with a barbell (95lb men/65lb women). A 400 m run followed by 15 overhead squats was one round. 

### 2.9. Statistical Analysis

Preliminary analyses were conducted to ensure no violation of the assumptions of normality, linearity, multicollinearity, and homoscedasticity. Normality was assessed using the Kolmogorov–Smirnov Statistic and normal P–P plots, linearity using scatterplots, multicollinearity from values of Tolerance and variance inflation factor, and homoscedasticity using a scatterplot of standardized residuals and predicted values. Outliers were inspected using a box plot and Cook’s distance. Descriptive statistics were performed on participant characteristics. Data analyses were performed using SPSS Version 25. Simple Pearson’s r correlations were used to determine association between CrossFit^®^ performance data and the physiological performance measures. For each CrossFit^®^ workout dependent variable, a stepwise multiple linear regression was created using significant correlative data. Significant variables that survived the regression analysis were used to create a predictive model of performance. Data are reported as means and standard deviations. The alpha level was set a priori at 0.05. 

## 3. Results

### 3.1. Preliminary Analysis

Preliminary analyses revealed two outliers and a violation in the assumption of normality. As previously mentioned, these two outliers were excluded from the analysis. Once removed, preliminary analyses were performed again, and all assumptions were met. 

### 3.2. Physiological Performance Data and Correlations

**Figure 1:**
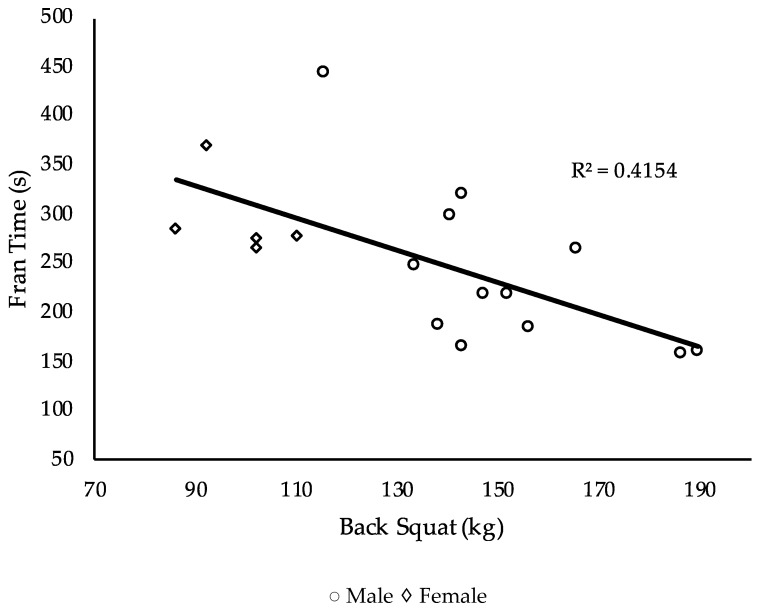
Plots of the relationship between back squat strength and Fran time.

**Figure 2:**
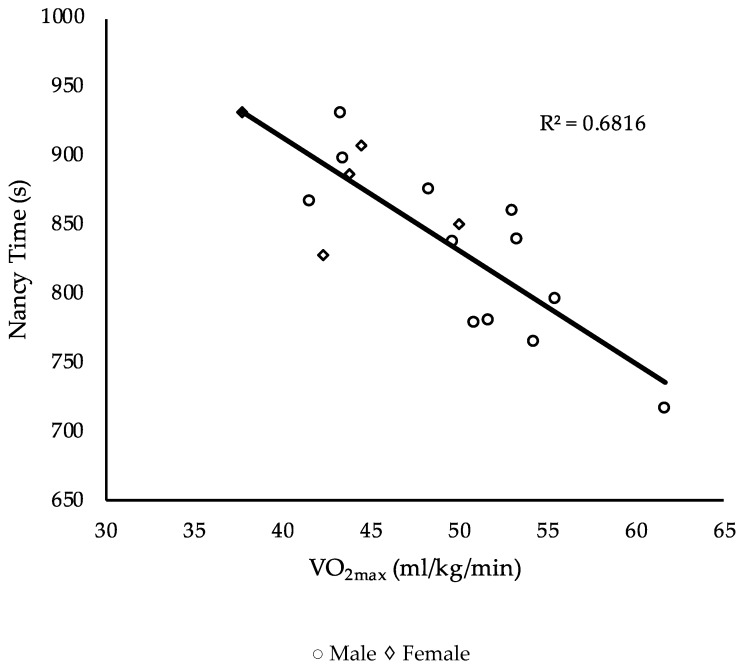
Plots of the relationship between VO_2max_ and Nancy times.

**Figure 3:**
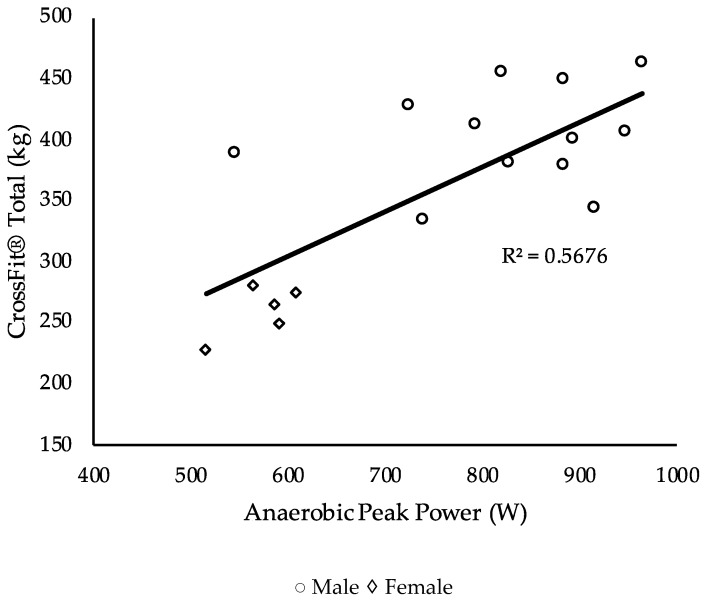
Plots of the relationship between anaerobic peak power and CrossFit^®^ total weight lifted.

Performance measurements are displayed in Table 2. The relationship between the independent (performance measures) and dependent (benchmark workouts) variables was investigated using a Pearson product–moment correlation coefficient. Results are displayed in Table 3. A stepwise multiple regression was used to assess the ability of the independent variables to predict the dependent variables. Results for each benchmark workout are displayed in Table 4, Table 5 and Table 6 and Figure 1, Figure 2 and Figure 3.

### 3.3. Fran Regression Model

For the workout Fran, back squat strength explained 42% of the variance in the model and was the only statistically significant measure, F (1, 15) = 10.7, *p* = 0.005. 

### 3.4. Nancy Regression Model

For the workout Nancy, VO_2max_ explained 68% of the variance in the model and was the only statistically significant measure, F (1, 15) = 32.12, *p* < 0.001. 

### 3.5. CrossFit^®^ Total Regression Model

Though the CrossFit^®^ total was used as a performance measure, it is also considered a benchmark workout, which is why it was included in the analysis [29]. Anaerobic peak power explained 57% of the variance and was the only statistically significant measure, F (1, 15) = 19.7, *p* < 0.001. 

## 4. Discussion

### 4.1. Summary

Results did not demonstrate CS and total body strength to be significant indicators of CrossFit^®^ performance but that different physiological measures predict performance better than others for specific CrossFit^®^ benchmark workouts. Moderate to strong correlations existed with many of the physiological measurements and the CrossFit^®^ benchmark workouts. The significant negative correlations demonstrated an inverse relationship between the independent and dependent variable, whereas positive correlations demonstrated a direct relationship. For instance, a significant negative correlation was seen between VO_2max_ and time to completion on the workouts Fran and Nancy, indicating a lower time and faster completion were observed by those with superior aerobic fitness as measured by VO_2max_. A significant direct relationship was observed between anaerobic peak power and the CrossFit^®^ total, indicating a higher anaerobic peak power is related to higher values obtained on the CrossFit^®^ total. Nevertheless, few variables survived the regression analyses for each workout. For Fran, back squat strength explained 42% of the variance. For Nancy, VO_2max_ explained 68% of the variance. Though the CrossFit^®^ total was used as a measurement of total body strength, it is also a common CrossFit^®^ benchmark workout and so was included in the analysis, in which anaerobic peak power explained 57% of the variance. Lastly, no significant correlations were demonstrated by physiological variables for the workout Grace.

### 4.2. Predictors of CrossFit^®^ Benchmark Performance

In this study, the wide variety of performance measures that best predicted performance demonstrated that no single variable predicted overall CrossFit^®^ performance across multiple workouts, though no significant correlations were demonstrated between physiological variables and the workout Grace. This was surprising, as strength measured from the CrossFit^®^ total has previously predicted performance on this workout, and back squat strength has been demonstrated to explain 87% of the variance for 1RM clean and jerk performance [14,30]. Further, in a study examining strongman performance on a log clean and press for as many reps as possible in 60 s, similar to 30 clean and jerks for time were performed during Grace, strength measured by a 1RM bench press significantly correlated to the number of repetitions performed (r = 0.76) [31]. Given that the average time to completion on Grace was approximately three minutes and can be performed without a squat, this may explain why strength did not influence Grace performance. However, the average CrossFit^®^ total score was approximately 40 kilograms less than in the study presented by Butcher et al. (2015). This may indicate that training status of the athlete influences performance. However, further research should explore the relationship between physiological variables and Grace, as no physiological variable demonstrated a relationship to performance on this workout.

Though total body strength was not a significant predictor of overall CrossFit^®^ benchmark performance, back squat strength was a significant indicator for the workout Fran. Fran requires participants to complete forty-five front squats, requiring both muscular strength and endurance as well as the ability to perform under fatigued conditions. Muscular strength and endurance have previously demonstrated significant relationships to one another [32,33]. Greater lower body strength has also demonstrated a significant relationship to performance under fatigued conditions in rugby players (r = 0.72, *p* = 0.013) [34]. Lower body strength, specifically squat strength, may influence other fast paced CrossFit^®^ workouts that require a squatting movement.

Previous literature recommended future research examine the workout Nancy, as data from this workout may better reflect physiological data collected during a running VO_2max_ test as were implemented within this study [14]. Given that CS is a maximal sustainable pace, and due to its strong relationship to VO_2max_, it was hypothesized to be a significant indicator of overall CrossFit^®^ benchmark performance [17,35]. Both CS and VO_2max_ were significantly correlated to Nancy performance, and both variables have been used to predict running performance [26,36]. However, VO_2max_ was the greatest predictor of performance on this workout, explaining 68% of the variance. This may indicate that those with a higher VO_2max_ may perform better on longer workouts that require running.

The CrossFit^®^ total was implemented as a measurement for total body strength. However, given that the CrossFit^®^ total has been used as a benchmark workout, further analysis was conducted to determine performance on the CrossFit^®^ total [29]. After excluding results from the squat, strict press, and deadlift, findings revealed that anaerobic peak power significantly explained 57% of the variance. Significant relationships between power and strength have previously been described [37,38,39]. Maximum power during the double leg press was significantly related to the 1RM achieved on the double leg press (R^2^ = 0.584, P < 0.001) [37]. A strong relationship between squat strength and peak power output from a counter movement vertical jump (r = 0.92) and static vertical jump (r = 0.93) has also previously been displayed [39]. Though these studies did not assess peak power using a WAnT, as in this study, previous findings have exhibited significant (*p* < 0.05) positive rank correlations between the counter movement jump and peak and mean power from the WAnT (τ = 0.59 and 0.76) [38]. These findings link leg strength to anaerobic peak power, which may explain the relationship of performance on the CrossFit^®^ total to anaerobic peak power.

No other study has displayed various significant indicators of CrossFit^®^ performance across multiple workouts as in the present study. Physiological performance measures predicted CrossFit^®^ performance, though results differed from previous studies. Butcher et al. (2015) revealed several significant correlations; however, only total body strength derived from the CrossFit^®^ total survived the regression analysis in predicting Fran and Grace performance. Total body strength did not survive the regression analysis in our study, only back squat strength for the workout Fran. Bellar et al. (2015) also examined various indicators of CrossFit^®^ performance, finding greater CrossFit^®^ experience, aerobic capacity, anaerobic power, and lower age to result in a significant model from a multiple linear regression on a workout. However, this study did not examine strength, and the workouts were not standardized CrossFit^®^ benchmark workouts. The utilization of benchmark workouts is important, as these are workouts that CrossFit^®^ professionals use to assess fitness levels, which have specific loading parameters and movement standards. Findings from both the current study and Butcher et al. (2015) revealed a strength measure to influence CrossFit^®^ performance on a CrossFit^®^ benchmark workout, indicating some consistency in findings. However, further investigation on CrossFit^®^ sport performance on other workouts is warranted. 

### 4.3. Limitations and Future Research

This study was not without limitations. The study consisted of seven days of data collection in which participants exercised at high and maximal intensities. Given the volume of visits and scheduling, data collection took place over the course of four weeks, and participants did not all perform the tests and workouts in the same order. Participants were not asked to refrain from training or recreational activities, aside from the 48 h prior to data collection, which affected sample size due to injuries during those outside activities. It is also possible that over the course of four weeks, physiological adaptations occurred. Equipment limitations also led to a reduced sample size, as pacing during the 3MT was not observed until too late and the cycle ergometer used for the WAnT could only provide enough resistance for someone weighing 94 kg. 

Future research may examine sex differences in benchmark workout performance. Another limitation of this study was that the sample size was not large, nor evenly distributed between males and females, to examine if differences between sexes existed. Sex performance differences have been previously displayed between military soldiers [40]. Females displayed significantly greater flexibility (*p* < 0.01) and better balance (*p* ≤ 0.001) compared to males [40]. However, males demonstrated significantly greater strength (*p* ≤ 0.001), aerobic capacity (47.5 ± 7.6 vs. 40.3 ± 5.4 mL/kg/min, *p* < 0.001), anaerobic power (13.3 ± 2.1 vs. 9.5 ± 1.7 W/kg, *p* < 0.001), and anaerobic capacity (7.8 ± 1.0 vs. 6.1 ± 0.8 W/kg, *p* < 0.001) compared to females [40]. It is important to note that in this study, males and females were tested under similar conditions [40]. In the current study, females performed the workouts at different weights than males. For this reason, it was assumed there would be no sex bias of performance. This is substantiated as male and female performance on thrusters and kettle bell swings prior to and after a 16-week high-intensity functional training program at a CrossFit^®^ gym did not differ, and males and females used different weights for these movements [41]. Further examination of the male and female population in this study revealed a discrepancy in experience. However, a Pearson’s r correlation revealed no significant correlation (*p* > 0.05) between experience and workout performance, indicating that experience would not be a significant predictor of performance. Nevertheless, this is just one study, and future research should investigate sex performance differences on CrossFit^®^ performance.

## 5. Conclusions

In conclusion, findings indicated that different physiological measures predicted performance better than others for specific CrossFit^®^ benchmark workouts. Back squat strength was the most significant indicator of Fran performance, suggesting athletes with a stronger lower body may be able to maintain a fast pace with less fatigue, completing the workout more quickly. VO_2max_ was the only significant predictor of Nancy performance, indicating athletes with a higher aerobic power would perform better on this workout. Lastly, performance on the CrossFit^®^ total revealed anaerobic peak power to be a significant indicator of total body strength, suggesting a combination of both strength and power may prove optimal in heavy workouts. Findings suggest that lower body strength, peak anaerobic power, and aerobic power may all prove favorable when competing in CrossFit^®^. Given the array of physiological variables that best indicate performance on various CrossFit^®^ workouts, athletes should ensure training to improve lower body strength and power as well as aerobic power.

## Figures and Tables

**Table 1:** Participant characteristics.

Participant Characteristics	All	Males	Females
N	17	12	5
Age (years)	28.0 ± 5.0	29.0 ± 5.6	25.6 ± 2.3
Height (cm)	173.8 ± 9.1	163.2 ± 49.7	166.4 ± 6.1
Weight (kg)	78.8 ± 9.8	83.5 ± 6.5	67.5 ± 6.5
Body Fat (%)	15.5 ± 4.9	13.3 ± 3.8	20.8 ± 3.0
CrossFit^®^ Experience (months)	43.2 ± 29.4	49.0 ± 32.7	29.4 ± 13.8

Note: The values are expressed as mean ± standard deviation (SD).

**Table 2:** Performance data.

Performance Data	All	Males	Females
N	17	12	5
VO_2max_ (ml/kg/min)	48.6 ± 6.2	50.6 ± 5.8	43.7 ± 4.4
CS (m/s)	3.52 ± 0.5	3.6 ± 0.5	3.3 ± 0.2
D′ (m)	220.5 ± 60.2	222.4 ± 59.8	216.0 ± 67.9
Anaerobic Peak Power (W)	753.3 ± 155.6	828.7 ± 116.9	572.4 ± 35.3
Anaerobic Mean Power (W)	571.8 ± 114.8	626.8 ± 85.4	439.8 ± 42.2
Fatigue Index (%)	43.7 ± 9.7	44.5 ± 8.5	41.9 ± 13.0
CrossFit^®^ Total (kg)	360.6 ± 80.0	402.6 ± 41.2	259.7 ± 20.9
Back Squat (kg)	135.5 ± 30.6	151.0 ± 21.2	98.5 ± 9.4
Strict Press (kg)	64.7 ± 15.3	72.7 ± 9.5	45.5 ± 5.1
Deadlift (kg)	160.3 ± 33.8	178.8 ± 18.6	115.8 ± 9.6
Fran (s)	254.8 ± 77.6	237.9 ± 83.9	295.4 ± 42.8
Grace (s)	173.4 ± 37.2	171.6 ± 38.0	177.6 ± 39.2
Nancy (s)	843.5 ± 61.2	827.9 ± 62.4	881.0 ± 42.2

Note: The values are expressed as mean ± standard deviation (SD). Abbreviations: VO_2max_, maximal oxygen consumption; CS, critical speed; D′, distance capacity for running speeds above CS.

**Table 3:** Correlations between CrossFit^®^ benchmark performance and physiological measures.

Performance Correlation Data	Fran (s) *n* = 17	Grace (s) *n* = 17	Nancy (s) *n* = 17	CF Total (kg) *n* = 17
VO_2max_ (ml/kg/min)	−0.562 * p = 0.019	−0.297 *p* = 0.247	−0.826 ** p = 0.000	0.505 * p =0.039
CS (m/s)	−0.568 * p = 0.017	−0.303 *p* = 0.237	−0.670 ** p = 0.003	0.364 *p* = 0.151
D′ (m)	0.104 *p* = 0.690	0.319 *p* = 0.211	0.156 *p* = 0.549	0.079 *p* = 0.763
Anaerobic Peak Power (W)	−0.317 *p* = 0.216	−0.250 *p* = 0.333	−0.634 ** p = 0.006	0.753 ** p = 0.000
Anaerobic Mean Power (W)	−0.399 *p* = 0.113	−0.251 *p* = 0.330	−0.679 ** p = 0.003	0.714 ** p = 0.001
Fatigue Index (%)	0.256 *p* = 0.322	0.120 *p* = 0.645	0.073 *p* = 0.782	0.147 *p* = 0.572
CrossFit^®^ Total (kg)	−0.599 * p = 0.011	−0.371 *p* = 0.143	−0.550 * p = 0.022	N/A N/A
Back Squat (kg)	−0.644 ** p = 0.005	−0.337 *p* = 0.185	−0.534 * p = 0.027	0.954 ** p = 0.000
Strict Press (kg)	−0.620 ** p = 0.008	−0.375 *p* = 0.138	−0.574 * p = 0.016	0.928 ** p = 0.000
Deadlift (kg)	−0.484 * p = 0.049	−0.360 *p* = 0.156	−0.495 * p = 0.044	0.966 ** p = 0.000

Note: * significant correlation *p* < 0.05; ** significant correlation *p* < 0.01; Abbreviations: VO_2max_, maximal oxygen consumption; CS, critical speed; D′, distance capacity for running speeds above CS.

**Table 4:** Summary of multiple regression analysis for Fran.

Variable	B	SE_B_	β	Observed Power
Back Squat (kg)	−1.634	0.50	−0.644 **	0.84

Note: ** *p* < 0.01; B = unstandardized regression coefficient; SE_B_ = standard error of the coefficient; β = standardized coefficient; observed power = post-hoc power analysis.

**Table 5:** Summary of multiple regression analysis for Nancy.

Variable	B	SE_B_	β	Observed Power
VO_2max_	−8.154	1.44	−0.826 **	0.99

Note: ** *p* < 0.01; B = unstandardized regression coefficient; SE_B_ = standard error of the coefficient; β = standardized coefficient; observed power = post-hoc power analysis.

**Table 6:** Summary of multiple regression analysis for CrossFit^®^ total.

Variable	B	SE_B_	β	Observed Power
Anaerobic Peak Power (W)	0.368	0.083	−0.753 **	0.97

Note: ** *p* < 0.01; B = unstandardized regression coefficient; SE_B_ = standard error of the coefficient; β = standardized coefficient; observed power = post-hoc power analysis.
